# Development of a Reference Transcriptome and Identification of Differentially Expressed Genes Linked to Salt Stress in Salt Marsh Grass (*Sporobolus alterniflorus*) along Delaware Coastal Regions

**DOI:** 10.3390/plants13142008

**Published:** 2024-07-22

**Authors:** Antonette Todd, Ketaki Bhide, Rita Hayford, Vasudevan Ayyappan, Mayavan Subramani, Lathadevi Karuna Chintapenta, Jyothi Thimmapuram, Gulnihal Ozbay, Venu (Kal) Kalavacharla

**Affiliations:** 1 College of Agriculture, Science and Technology, Delaware State University, Dover, DE 19901, USA; ritakusiappiah@gmail.com (R.H.); vasudevanbiotech@gmail.com (V.A.); mayavans@gmail.com (M.S.); gozbay@desu.edu (G.O.); venukalav@gmail.com (V.K.); 2 Bioinformatics Core, Purdue University, West Lafayette, IN 47907, USA; k2bhide@gmail.com (K.B.); jyothit@purdue.edu (J.T.); 3 Department of Biology, College of Arts and Sciences, University of Wisconsin River Falls, River Falls, WI 54022, USA; lathadevi.chintapenta@uwrf.edu

**Keywords:** transcriptome, next-generation sequencing, marsh grass

## Abstract

Salt marsh grass (*Sporobolus alterniflorus*) plays a crucial role in Delaware coastal regions by serving as a physical barrier between land and water along the inland bays and beaches. This vegetation helps to stabilize the shoreline and prevent erosion, protecting the land from the powerful forces of the waves and tides. In addition to providing a physical barrier, salt marsh grass is responsible for filtering nutrients in the water, offering an environment for aquatic species and presenting a focal point of study for high salt tolerance in plants. As seawater concentrations vary along the Delaware coast from low to medium to high salinity, our study seeks to identify the impact of salt tolerance in marsh grass and to identify genes associated with salt tolerance levels. We developed more than 211,000 next-generation-sequencing (Illumina) transcriptomic reads to create a reference transcriptome from low-, medium-, and high-salinity marsh grass leaf samples collected from the Delaware coastline. Contiguous sequences were annotated based on a homology search using BLASTX against rice (*Oryza sativa*), foxtail millet (*Setaria italica*), and non-redundant species within the Viridiplantae database. Additionally, we identified differentially expressed genes related to salinity stress as candidates for salt stress qPCR analysis. The data generated from this study may help to elucidate the genetic signatures and physiological responses of plants to salinity stress, thereby offering valuable insight into the use of innovative approaches for gene expression studies in crops that are less salt tolerant.

## 1. Introduction

Soil and water salinization are among the global challenges limiting the growth and production of crop plants [1]. Elevated salinity levels can significantly impact plants and animals, particularly in communities that are located near sea water and in environments with high concentrations of salt. The sustainability of terrestrial animals can be negatively influenced by salinity [2], unlike the case for aquatic animals such as fish, crabs, and waterfowl that are acclimated to this environment. Additionally, higher salt concentrations could severely impact plant life that grows in these shoreline regions [3]. Furthermore, the use of irrigation systems in farming for watering practices may affect plants by the quantity of nutrients introduced to them, having a downstream effect on the surrounding environment. Excess nutrients, such as nitrogen and phosphorus, can be carried through runoff and land in nearby waterways, lakes, or groundwater, impacting unintended areas through eutrophication [4]. Due to the adverse effects of high salt concentrations, areas surrounding high-salinity volumes of water are less desirable in the planning and dedication to planting crops [5]. High salt concentrations can present challenges in many dietary staple crop species such as rice (*Oryza sativa*), which is the most salt-sensitive cereal crop [6,7,8], and is necessary for the sustainability of many populations across the globe; notably in the Southeast Asian region [9]. Here, increased salinity can account for up to 69% yield loss in some instances. An additional crop that can be impacted by high salt is the lima bean (*Phaseolus lunatus*), which is a staple grown in the state of Delaware. Salinity can negatively impact lima beans and cause physiological changes in lima beans exposed to increased levels of salt in water irrigation systems [10].

Transcriptome studies can help to reveal the genes and regulatory mechanisms associated with abiotic stresses such as salt stress by examining upregulation (increase in gene expression) and downregulation (decrease in gene expression) in response to stresses [11]. Over the past decades, transcriptome analyses have been a key factor in examining gene expression data for numerous organisms, including various plant species. Thale cress or mouse ear cress, more commonly known as Arabidopsis (*Arabidopsis thaliana*), is one of the earliest publicly available sequenced genomes and transcriptomes and still serves as a vital reference for comparative studies among plant species. Many of the publicly available sequence databases such as the National Center for Biotechnology Information (www.ncbi.nlm.nih.gov), the European Bioinformatics institute (www.ebi.ac.uk), and the Plant Comparative Genomics portal of the Department of Energy’s Joint Genome Institute (phytozome-next.jgi.doe.gov) include data assembled from Arabidopsis, as it is one of the easier plants to propagate and has a smaller genome. To this effect, several transcriptomic studies have been conducted using Arabidopsis as a reference including fungal-rust-challenged common bean (*Phaseolus vulgaris*) [12]; switchgrass (*Panicum virgatum*) under heat and drought stress [13]; and our current study in salt marsh grass, also identified as cord grass or smooth cord grass (*Spartina alterniflora*), naturally occurring under saltwater conditions.

Salt marsh grass (*S. alterniflora*), more recently known as *Sporobolus alterniflorus* [14], is a halophytic, salt-tolerant plant, growing in areas in which most plant species would be negatively impacted by the high salinity concentrations. Salt marsh grass thrives in seawater, which can be as salt concentrated as 3.5% (*w*/*w*) salinity [15]. Salt marsh grass can also act as a physical barrier along coast lines, useful for its restorative properties in areas of erosion [10] and for protection of the shoreline [16]. Salt marsh grass may also provide a living environment for some aquatic animal species such as turtles, birds, and fish, as well as serving as a key species to study salt tolerance. The salt marsh grass used in the current study grows in Delaware coastal regions where the salinity varies depending upon the location, with northern shorelines typically presenting a lower salt concentration. Areas in the Southern Delaware coastal regions tend to be more highly salt concentrated, which does not appear to have a negative physiological impact on the plant’s ability to flourish and develop. Because of this, salt marsh grass may offer insight into the plant’s ability to withstand high salinity levels, which will be critical as sea levels continue to rise [17]. In Li et al., 2018, the comparative effects of salinity stress on the marsh grass types *S. alterniflorus* and *Phragmites australis* compounded with water logging stress revealed negative effects on the photosynthetic process and shoot growth in *P. australis* but negligible effects on *S. alterniflorus*. Therefore, it can be determined that Sporobolus has a high salt tolerance; moreover, the high salinity can also serve as a major factor in its nutrient uptake and sequestration. In 2010, the Deepwater Horizon explosion and oil spill negatively impacted plants and wildlife in the vicinity of the spill. Interestingly, salt marsh grass that flourished near the region of the oil spill proved resilient in its growth [18]. This information may also be beneficial in exploring the impact of salinity and mitigating techniques on crops cultivated for human consumption [19]. Our capability to improve and maintain crop growth in adverse conditions such as global temperature changes and increased drought conditions has the capacity to be impactful to human survival. Although there are additional transcriptome studies identifying the differential expression of salinity genes in Sporobolus species or salt marsh grass [20,21], there is still room for the development of additional information on the *S. alterniflorus* species. Furthermore, our sample analyses were conducted using naturally occurring marsh grass plants derived from the uncontrolled environment. The samples used in this study give a snapshot of the environmental conditions in the Delaware coastal regions.

In this study, our key objectives are to (1) develop a comprehensive reference transcriptome for salt marsh grass from naturally growing samples collected from high (>25 ppt), medium (~15–20 ppt), and low (<5 ppt) salinity concentrations of water along the Delaware coastal regions, (2) identify differentially expressed genes that are associated with the salinity conditions derived from various salt concentrations in the environment in which the collected plants naturally grew, and (3) analyze the genetic basis of the salt stress response. In addition to identifying genes associated with salt stress tolerance, we identified Gene Ontology (GO) terms and pathways related to the salt stress response. Furthermore, molecular genetic analysis of the samples, used in conjunction with transcriptomic data gained from this study, may help in identifying genes that are associated with salinity tolerance such as nutrient-uptake-, desiccation-tolerance-, and drought-related genes. The goal of the current research is to identify the differential effects of various saltwater concentrations on salt marsh grass gene expression. We intend to identify the genes associated with a gradient salinity concentration by analyzing the transcriptomic sequence data derived from salt marsh grass tissues collected along the Delaware coastline. Information gained from this study, such as differentially expressed genes (DEGs), functional enrichment, and identification of Kyoto Encyclopedia of Genes and Genomes (KEGG) categories, may help in elucidating the beneficial and unfavorable effects of salinity tolerance and susceptibility in other related crops that are used for human consumption such as rice and millet.

## 2. Results

### 2.1. Marsh Grass Transcriptome Assembly

A salt marsh grass transcriptome final assembly consisting of a total of 211,808 contiguous sequences (contigs) was generated from a quality-trimmed RNA-Seq sequence derived using concatenated assemblies of Trinity [22], Transabyss, and SOAPdenovo-Trans [23] to align the RNA-Seq data. The contigs generated were also evaluated using BUSCO (v 4.0.6) [24]. The final assembly used RNA-Seq data generated using samples from all the tissues collected from the three sampling regions including high-, medium-, and low-salinity samples. The assembled transcriptome was annotated using three publicly available reference databases that consist of basic local alignment search tool (BLAST) [25] results from Viridiplantae nr (non-redundant), foxtail millet (*Setaria italica*), and rice (*O. sativa*) protein databases. The Gene Ontology (GO) and Kyoto Encyclopedia of Genes and Genomes (KEGG) resources provided information from rice and foxtail millet databases. The differential expression analyses were performed among high-, medium-, and low-salinity salt marsh grass tissue samples using DESeq2 (differential expression seq) and EBSeq (empirical Bayesian seq) based on the expected counts generated by RNA-Seq by expectation maximization (RSEM) (v 1.3.3).

### 2.2. BLAST Summary Statistics

The final set of contigs was annotated using BLASTX against the Viridiplantae nr database, accessible through NCBI. The BLAST results were generated using sequence data from the collected salt marsh grass samples. The contigs were also annotated against the rice (*O. sativa*) and foxtail millet (*S. italica*) reference transcriptomes using BLASTX, which are detailed in Table 1. Of the 211,808 contigs generated, 186,170 (88%) aligned to at least one of the three databases. Only queries with alignment E-values of less than 10 × 10^−3^ were retained and included in the results file. Of the total amount of contigs, 157,700 (74.5%) aligned to all the three databases. The BLAST hits were further characterized by unique hits for the Viridiplantae nr database, the rice proteins database, and the foxtail millet proteins database identifying 61,699, 23,982, and 23,358 unique hits, respectively.

### 2.3. Number of Differentially Expressed Contigs from Each Method

**Figure 1:**
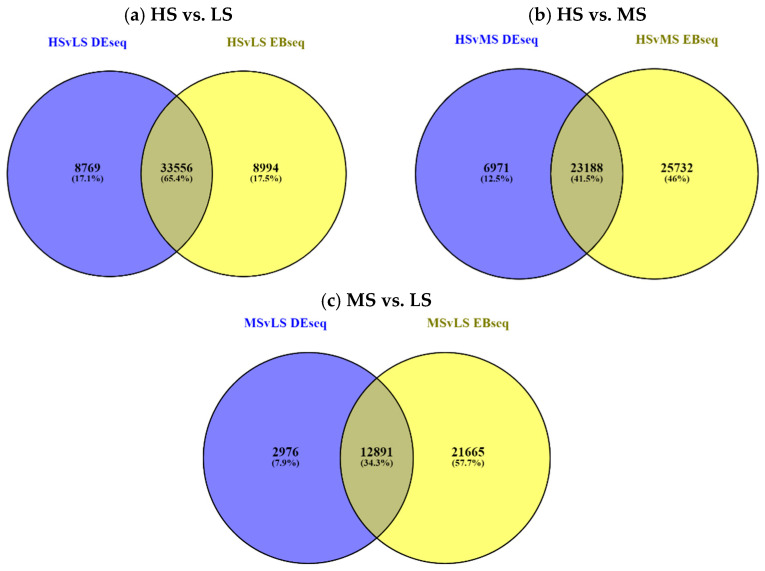
Venn diagram of differentially expressed (DE) contigs under salt conditions detected by two methods (DESeq2 and EBSeq). The Venn diagrams show common and unique DE contigs between these methods.

Comparative analyses were performed to identify differential expressions among the three sample types including the high-salinity biological replicate (HS), the medium-salinity biological replicate (MS), and the low-salinity biological replicate (LS). The differential expressions were detected and measured using two methods, DESeq2 (v 1.28.1) [26] and RSEM (v 1.3.3) [27]. Comparisons between HS vs. MS, HS vs. LS, and MS vs. LS resulted in the identification of 30,159 DESeq2 and 48,920 RSEM-EBSeq, 42,325 DESeq2 and 42,550 RSEM-EBSeq, and 15,867 DESeq2 and 34,556 RSEM-EBSeq, respectively. The differentially expressed genes were identified using the following criteria: log2FC ≥ 2 for upregulated DEGs and ≤−2 for downregulated DEGs at a *p*-value < 0.05. The number of differentially expressed contigs are listed in Table 2 and visualized in Venn diagram format in Figure 1.

### 2.4. Number of Differentially Expressed Contigs from EBSeq with Multiple Conditions

In total, four patterns were generated from the EBSeq method with a false discovery rate (FDR) of ≤0.05 or a posterior probability differentially expressed (PPDE) of ≥0.95. The patterns categorize and distinguish expression similarity among the high (HS)-, medium (MS)-, and low (LS)-salinity contig data generated from each sample (Table 3). Pattern 1 consists of contigs that have similar expression between HS and MS but are different for LS. Pattern 2 consists of contigs that have similar expressions between HS and LS but are different for MS. Pattern 3 consists of contigs that have similar expression between MS and LS but are different for HS. Pattern 4 consists of contigs for which there is different expression between all conditions.

### 2.5. Gene Expression Analysis

#### Gene Identification and Primer Design

**Figure 2:**
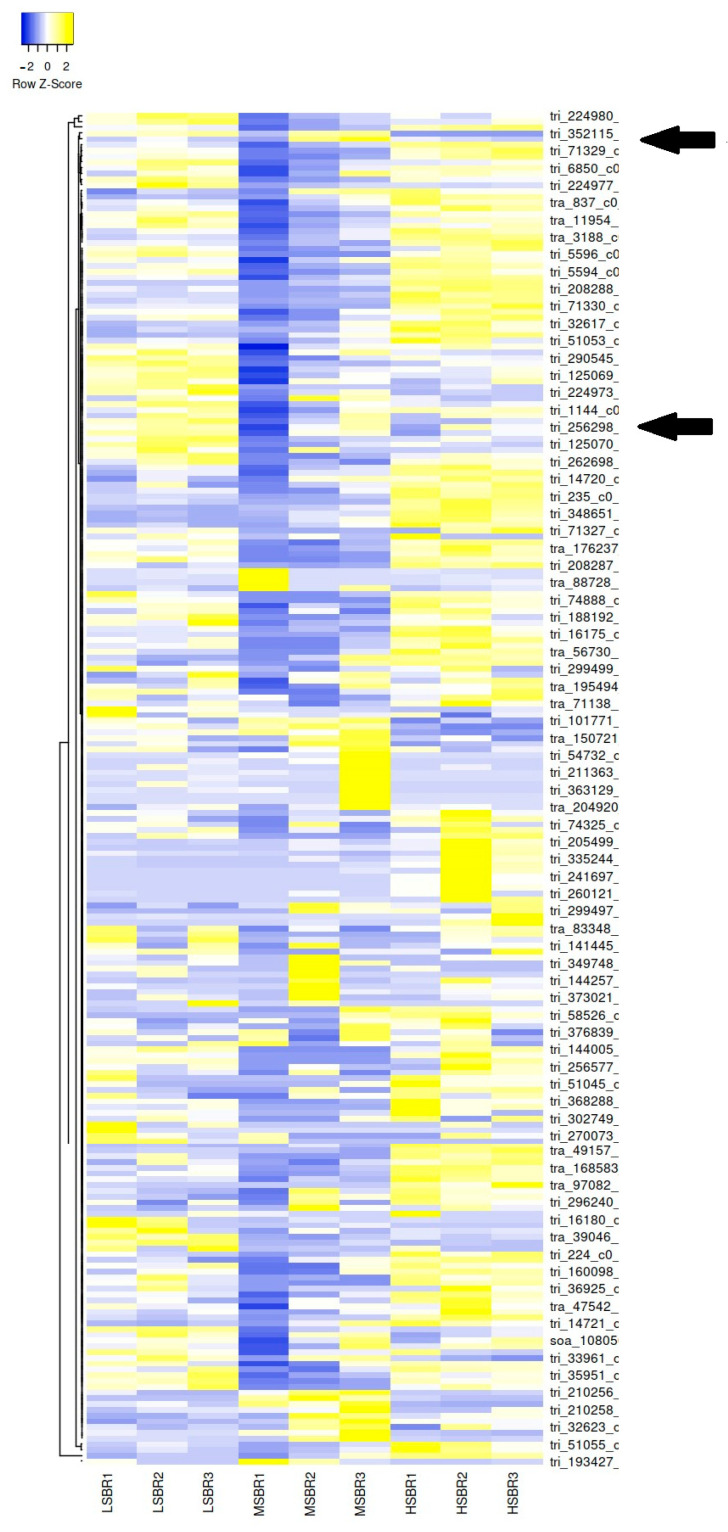
Heatmap derived from salt-related contigs identified from multiple replicate samples in low-, medium-, and high-salinity tissue collections. Two of the three contigs used to design primers for qPCR are identified.

To identify differential gene expression, data were derived from HS, MS, and LS, samples with a focus on BLAST descriptions related to salinity. The BLAST hit descriptions for non-redundant Viridiplantae, foxtail millet, and rice were sorted to select descriptions that included sodium, salt, and salinity. Keywords related to salt were selected as this was a major focus of the study, and we anticipated differential expression to be most apparent in these categories. Of approximately 211 K contiguous sequences, 400, 394, and 373 samples were identified for HS vs. LS, HS vs. MS, and MS vs. LS, respectively. A heatmap was generated using the expression data from three biological replicates (low, medium, high salinity) including three technical replicates for each sample (www.heatmapper.ca) (Figure 2). Along with the expression data visualized in the heatmap, several PCR primers were designed from select contig ID numbers associated with BLAST hit descriptions linked to salt, sodium, or salinity and showing differential expression across the three salt concentration collection types. Additionally, the contigs were BLASTed to the Phytozome (phytozome-next.jgi.doe.gov) database and identified *Oropetium thomaeum*, a small-sized (245 mb) model plant that is associated with desiccation tolerance [28].

### 2.6. qPCR Analysis

Quantitative real-time PCR (qPCR) was performed to identify differential gene expression among the selected targets associated with salt-related contigs derived from the sequence data and chosen from rice, foxtail millet, and the small desiccation-tolerant plant (*Oropetium thomaeum*). The primers designed for the qPCR analysis were used to distinguish differential expression among the salinity sample types. Both rice and foxtail millet were included as references in the original mapping of the marsh grass transcriptome sequences. Additionally, Oropetium was used as a reference because of its similarity to marsh grass as well as its small genome size of 250 mb and nine chromosomes (Phytozome.org).

**Figure 3:**
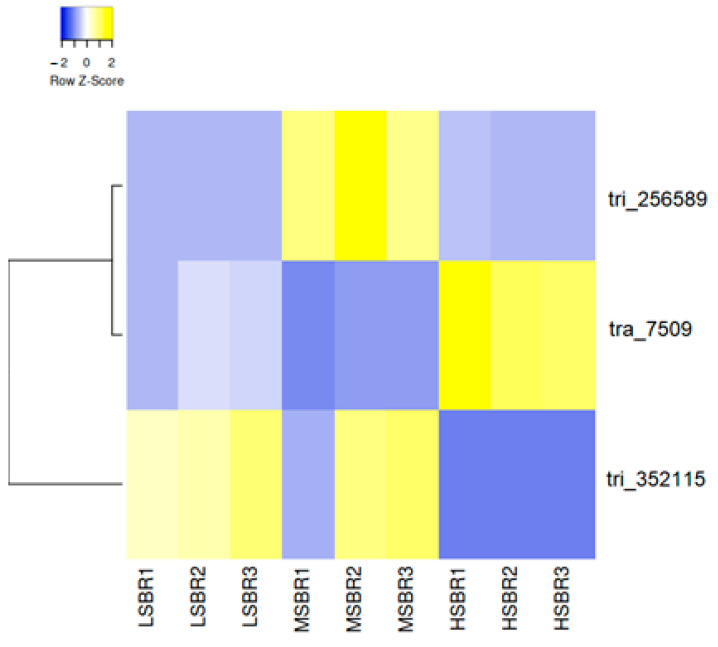
Heatmap derived from three selected contigs identified as having differential expression among low-, medium-, and high-salinity samples. The tri and tra labels are annotation IDs generated for contigs when developing the transcriptome. The prefix tri is associated with the Trinity assembly tool, and tra is associated with the Transabyss assembly tool.

**Figure 4:**
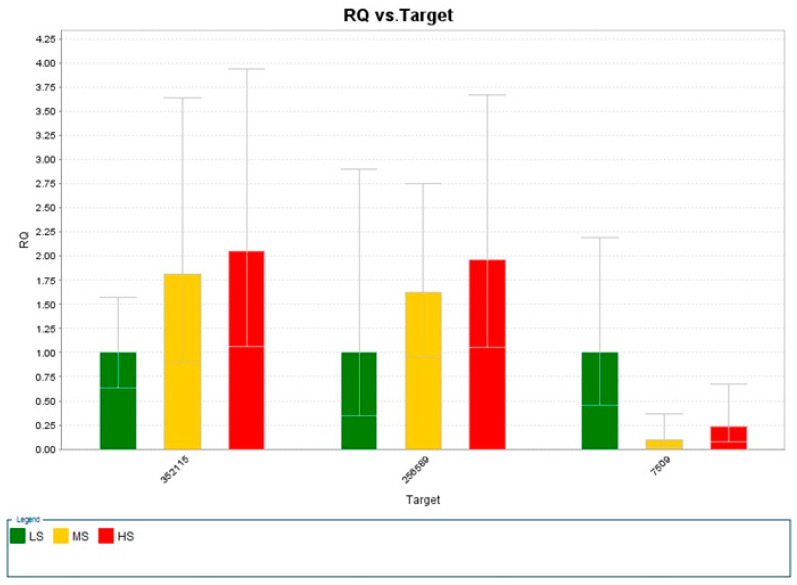
qPCR expression analysis across three contig-derived primer sets for each salinity level including low (LS)-, medium (MS)-, and high (HS)-salinity samples. Error bars for each sample were calculated using the relative quantifications (RQ Min and RQ Max) associated with the endogenous control (18s rRNA).

Three primers sets were designed from the contigs derived from the original transcriptome assembly sequences in Figure 3 and Table 4, while seven others were designed from the genes relating to salt expression (Supplementary Table S1). The real-time qPCR analysis results indicate insignificant differential expression across salinity sample concentrations Figure 4.

Ten total primer sets (three sets for results from Figure 4 and Table 5) were designed for the qPCR analysis. The primer sets were designed from salt-related genes and contigs using comparative analysis between two samples, i.e., HS vs. LS, etc. The primers were designed from genes related to a calcium/sodium exchanger protein in foxtail millet and a cation exchange gene in rice (Ca^2+^/H^+^ antiporter VCX1 and related proteins). In the non-redundant Viridiplantae database, the sequence is associated with hypothetical protein signatures in green foxtail (*Setaria viridis*) and weeping lovegrass (*Eragrostis curvula*).

### 2.7. Functional Categorization of the DE Genes (Enrichment Analysis)

A gene enrichment analysis was conducted to better interpret the gene expression data for upregulated and downregulated salt genes. The most represented Gene Ontology (GO) term enriched in the DESeq contigs in the HS vs. LS and HS vs. MS samples was “sodium/calcium exchanger protein” [GO:0055085 and GO:0016021, respectively]. Other terms enriched in the unigenes included “transporter activity” [GO:0005215] and metal ion transmembrane transporter activity [GO:0046873].

### 2.8. Transcription Factors and KEGG Pathway Analysis

Transcription factors were identified using comparative analysis between each collection type. We further retrieved significant pathways of the DEGs from the Kyoto Encyclopedia of Genes and Genomes (KEGG) pathway database. The contig name (DEG_name) and Rice KEGG id with the respective pathway name comparison of HS vs. LS, MS vs. LS, and HS vs. MS are shown in Table 6.

## 3. Discussion

The focus of the reported study was to develop a reference transcriptome and to identify differential expression in salt-related genes in marsh grass samples collected from areas varying in salinity concentrations. We successfully developed a high-quality reference transcriptome utilizing environmental samples collected at Delaware Inland Bays and beaches that are representative of salt concentration gradients in the local region. Of the approximately 211 K contiguous sequences that were identified and annotated, more than 400 are recognized as being associated with salinity, salt, and sodium for one or more of the reference datasets used for comparison. Our BLAST summary statistics detailed in Table 1 identify non-redundant Viridiplantae, rice, and foxtail millet as being 75% or more aligned to the more than 200 K contigs. This demonstrates marsh grass’s similarity to foxtail millet and rice for this comparative study.

We employed two methods, DESeq2 and EBSeq, to identify differential expressions between sample types. Read mapping uncertainty is frequent among de novo assembled transcripts. To account for possible ambiguity, we used an empirical Bayesian (RSEM-EBSeq pipeline) analysis tool in addition to DESeq and presented the results in Table 2. and Figure 1. When the RSEM-EBSeq pipeline was run to find DE contigs for multiple conditions, additional files of “Patterns”, which define all expression patterns over the conditions, and “condition means”, which give normalized mean values for each transcript at each condition, resulted. These condition mean values were used to calculate the fold change (FC) and log2FC between the two conditions and are provided in Table 3.

Since the study sought to identify differential expression in salt-related genes, the focal point of the qPCR analysis centered on choosing contigs associated with gene expression in genes relating to salinity. In Figure 2, we created a heatmap dedicated to the sequence data derived from more than 400 contigs associated with salt-relatedness over the LS, MS, and HS samples. We selected three contigs (Table 4) using comparative expression analysis as the focal point to perform further analysis through qPCR and created a smaller heatmap dedicated to those data (Figure 3). Figure 4 presents our qPCR expression in each sample across the three targets. Contrary to the sequence data, we observed higher expression in the HS sample in targets 32115 and 256589. We also noted that there was variation among the MS replicate samples. This was apparent through visual observations of the overall 419-contig heatmap (Figure 2).

In addition to the targets designed from the contigs, we used BLAST analysis to identify other genes for qPCR analysis. Sequences were chosen from comparisons (HS vs. LS) exhibiting a higher than two log2 fold change from the differential gene expression files derived from transcriptome development. The chosen sequences were BLASTed against rice, foxtail millet, and other related species to identify candidate genes for target primer development. Again, we anticipated higher variability in the expression data because of the variation in the transcriptome-derived data. Table 5 details the comparative expression analysis for the contig-derived primers. The table details data for sample name, target name, RQ (relative quantification), RQMIN (RQ minimum), RQMAX (RQ maximum), CT (cycle threshold), CTSD (CT standard deviation), DCT mean, and DDCT.

Although we anticipated identifying differential expression among the samples mostly within the genes associated with salt, we found many genes with a differential expression. The comparative data from the three sample gradients including biological replicates indicate an overlap of expression across sample types. The transcriptome analysis revealed the regulation of genes for transcription factors (TFs) and stress-responsive proteins related to salt stress as well as other genes, such as those associated with the KEGG IDs identified in Table 6. However, there were several TFs that were unassociated with KEGG IDs, for example, the contig “tri_2672_c0_g1_i1” annotated as a basic helix–loop–helix (bHLH) DNA-binding superfamily protein transcription factor was differentially expressed in HS vs. LS, HS vs. MS, and MS vs. LS samples from both DESeq2 and EBSeq analyses. The contig had the best hit in the rice genome as LOC_Os04g41570.1 was downregulated in both HS vs. LS and HS vs. MS samples. Interestingly, the contig tra_67737_c0_g1_i1 with rice hit LOC_Os08g41320.1, also annotated as the bHLH transcription factor, was upregulated in salt marsh grass. Several TFs including bHLH have been identified to play a critical role in abiotic stresses such as drought, salinity, and cold stress [29].

The contig “tri_13190_c0_g1_i1” with rice hit LOC_Os02g08440.4 encoding WRKY DNA-binding protein 40 was upregulated in both HS vs. LS and HS vs. MS samples. Similarly, the contig “tri_247732_c0_g1_i1” with rice hit LOC_Os01g18584.1, annotated as a WRKY family TF, was also upregulated in MS vs. LS samples. WRKYs are significant family TFs associated with plant development, defense regulation, and stress responses [30]. The WRKY family of TFs has been identified to respond to salt stress. Interestingly, contrary to our findings, ZmWRKY*114* was downregulated in maize during salt stress [31]. In addition, ion transport genes such as NRAMP metal ion transporter family protein with the contig tri_244640_c0_g1_i1 and a rice hit LOC_Os07g06130.1 was upregulated more than two-fold under high salinity stress in HS vs. LS samples. This finding supports previous studies [20,21] on the expression of salt-sensitive genes. Our study has revealed not only salt-tolerant genes but also key genes and transcription factors involved in abiotic stresses associated with salt. The current study identifies the Gene Ontology (GO) signatures associated with salinity relatedness, characterizing salt and sodium biological processes. As mentioned previously, the two GO terms most represented in the analyzed samples were GO:0055085 and GO:0016021, which are annotated as “sodium/calcium exchanger proteins” in foxtail millet and as “cation exchanger” in rice. Additionally, the KEGG pathway annotation of the DEGs or contigs showed several stress-response pathways, such as phenylpropanoid biosynthesis, amino acid biosynthesis, photosynthesis, glutathione metabolism, and MAPK signaling pathways. These significant pathways have been similarly reported in a previous salt-response study [1,21] and in drought and heat stress [13].

As the focal point for the current study is the impact of sodium on salt marsh grass and its ability to tolerate salt stress, there is still room for detailed analyses and characterization of other nutrients and their effects on related plant species. In our qPCR analyses, we encountered variation among biological replicates from the same collection site and salinity profile. We found variations and juxtapositions between the transcriptomic and qPCR analyses that displayed conflicting gene expression values between samples. In essence, the environmental samples present unregulated influences from factors that are not related to salt stress or tolerance but may have an impact on downstream molecular analyses. Unregulated influences such as human interaction, accessibility, or uncontrolled rain events can have an impact on sample collection and variation. When collecting samples, we took great efforts to collect during periods in which rain events had not occurred for five or more days. Water samples were measured before collecting the marsh grass samples to ensure the salinity concentration was at a desired level. Future steps can include controlled treatments of related plant species under similar conditions to mimic the effects of salt on the physiological aspects and gene expression profiles. We can theorize that plant species related to marsh grass may exhibit similar characteristics under parallel salt gradients. The implications may be very beneficial to the planning and planting of staple crops in traditionally less desirable landscapes for the sake of sequestering more amounts of useful land. This will require additional research including plant growth, treatment, sequence analysis, candidate gene identification, and primer design for molecular analysis.

## 4. Material and Methods

### 4.1. Sample Collection

Salt marsh grass leaf tissue samples were collected from three Delaware coastal regions identified as having high (>25 parts per thousand, ppt), medium (15–20 ppt), and low (0–5 ppt) salinity and served as three biological replicates. The three regions included Rehoboth Bay (Rehoboth watershed) latitude: 38.62922° N; longitude: 075.0718° W, Cedar Creek (Mispillion River watershed), latitude: 39.04792° N; longitude: 075.39253° W, and Murderkill River, latitude: 39.01350° N; longitude: 075.46378° W, for high-, medium-, and low-salinity samples, respectively. Three technical replicates were collected for each of the three biological replicates. Three individual plants were collected from three different sites totaling nine samples (three from high salt, three from medium salt, and three from low salt concentration). The Samples were collected for each and transported to the laboratory for debris removal and further processing. The leaves were sorted to remove dry or brown leaf debris and briefly rinsed in type 2 Millipore water. Next, the samples were flash frozen in liquid nitrogen and stored in a −80 freezer for the subsequent steps. The samples were stored by biological replicate and technical replicate for each. Tissues from the technical replicates were ground with liquid nitrogen in a mortar and pestle and split into two batches: one for downstream RNA-Seq transcriptome analysis and one for ChIP-seq epigenome analysis.

### 4.2. Library Preparation and Sequencing

The total RNA was isolated for each technical replicate from the tissues aliquoted for transcriptome analysis by using the TRIzol reagent (Invitrogen, Waltham, MA, USA). RNA samples were quantified by Qubit (New York, NY, USA) and checked for quality using gel electrophoresis. The quantified RNA samples were normalized to 2 µg to achieve consistent concentrations across the samples. Sequence libraries were constructed for each sample using the TruSeq RNA Library Prep Kit v2 from Illumina (San Diego, CA, USA). The completed libraries were checked for successful library development by assessment on gel electrophoresis. Samples were delivered to the Delaware Biotechnology Institute at the University of Delaware for sequencing. The completed sequencing data were forwarded to the Bioinformatics Core at Purdue University for bioinformatic analysis.

### 4.3. Transcriptome Assembly

Sequence quality was assessed using FastQC (v 0.11.2) (https://www.bioinformatics.babraham.ac.uk/projects/fastqc/) (accessed 31 July 2020) for all the samples and quality trimming was performed using TrimGalore (v 0.6.4) (https://www.bioinformatics.babraham.ac.uk/projects/trim_galore/) (accessed 31 July 2020) to remove bases with a Phred33 score of less than 30 and the resulting reads of at least 100 bases were retained. The quality-trimmed reads were used to perform transcriptome assembly.

KmerGenie (v 1.6982) [32] was used to find the best possible kmer for assembly based on the quality-control reads. Kmer 67 was determined to be the best for performing transcriptome assembly.

The quality-trimmed reads were assembled using three tools—Trinity (v 2.10) [22], Transabyss (v 2.0.1), and SOAPdenovo-Trans (v 1.0.4) [23]. Assembly using Trinity was performed using the default parameters except for read normalization. Trinity does not have an option to change the kmer value, so the default kmer value of 25 was used. The SOAPdenovo-Trans and Transabyss assemblies were also performed using default parameters and kmer 67.

The contigs with 500 nucleotides or more from all the assembly programs were concatenated using the “merge assembly” script, which uses TransDecoder LongOrf (v 3.0.1) and cd-hit-est (v 4.8.1) [33]. This script has the following parameters: 100 m, i.e., at least 100 amino acids peptide size after running TransDecoder will be retained, and aS 1.00, i.e., an alignment coverage threshold for shortest sequence will be used for cd-hit clustering.

### 4.4. Differential Expression Analysis

The quality-trimmed reads were mapped against the transcriptome using bowtie2 (v 2.3.5.1) [34] with default parameters. RSEM (RNA-Seq by expectation maximization) (v 1.3.3) [27] within the Trinity plugin was used to estimate transcript abundance at the gene level. RSEM generates a normalized measure of transcript expression that considers the transcript length, the number of mapped reads to the transcript, and the total number of reads that mapped to any transcript. RSEM also reports other normalized values such as “fragments per kilobase transcript length” (FPKMs) and “transcripts per million transcripts” (TPMs). These estimated fragment counts (expected counts) at the gene level (based on RSEM) are used by DESeq2 and EBSeq to find differentially expressed contigs. RSEM-EBSeq (rsem-run-ebseq script) can find DE (differentially expressed) contigs for two conditions (e.g., HS vs. MS) at a time as well as find DE contigs for multiple conditions (HS, MS, and LS) at once. DE contigs at a false discovery rate (FDR) of ≤0.05 can be obtained using the rsem-controlfdr script. When the RSEM-EBSeq pipeline was run to find DE contigs for multiple conditions, additional files of “Patterns”, which define all expression patterns over the conditions, and “condition means”, which give normalized mean values for each transcript at each condition, were compiled. These condition mean values were used to calculate the log (base 2) fold change (log2FC) and fold change (FC) between the two conditions. The result files from the two-condition testing and the multiple-condition testing are provided in the Supplementary Materials. Differential expression analysis among the samples was conducted using “R” (Version 4.0.2). A basic exploration of the expected counts generated from RSEM, such as accessing data range and library sizes, was performed to ensure data quality. DESeq2 (v 1.28.1) [26] was used to identify differentially expressed features, in which normalized factors were calculated and gene-wise dispersion was estimated. Empirical Bayes shrinkage was also applied for the dispersion estimation. In DESeq2, differences between the negative binomial distribution of counts for the two experimental conditions were evaluated to identify differential gene expression (DGE) with *p*-value and adjusted *p*-values of FDR to correct for multiple tests. The differentially expressed contigs detected by the two methods (DESeq2 and EBSeq) were compared in a Venn diagram created using Venny (http://bioinfogp.cnb.csic.es/tools/venny) (accessed 1 February 2023). The Venn diagrams and common and unique differentially expressed contig IDs between these methods are provided here.

The resulting sequences were selected by salt/sodium/salinity relatedness and analyzed for upregulation vs. downregulation by two-way comparison between the samples. Log2 fold change values were sorted from highest to lowest to determine upregulation vs. downregulation. For example, in the Excel datasheet “MVLDESEQ”, medium-salinity samples vs. low-salinity samples are compared and sorted by log2 fold change to determine gene expression values. This was carried out for all the comparative datasets including “HVLDESEQ”, which is high vs. low, and “HVMDESEQ”, which is high vs. medium.

### 4.5. cDNA Preparation

Complementary DNA (cDNA) samples were produced for qPCR analysis using ProtoScript^®^ II First Strand cDNA Synthesis Kit (New England Biolabs, Ipswich, MA, USA) following the manufacture’s protocol. Each sample began with 1 μg of total RNA and was quantified with NanoDrop after cDNA conversion. In addition to reverse-transcribed RNA, a no-template water control and a no-enzyme control were both included.

### 4.6. qPCR Analysis

The real-time quantitative polymerase chain reaction (qPCR) was performed using Applied Biosystems™ 2X SYBR™ Green PCR Master Mix (Thermo Fisher Scientific, Waltham, MA, USA). We followed a protocol published in *Nature Protocols* for obtaining relative quantification using the DDCT method [35]. An Applied Biosystems 7500 Real-Time PCR system was used to perform qPCR analysis with primer sets and samples. For each qPCR analysis, samples were performed in triplicate for each LS, MS, and HS in addition to a water control. In addition to the target gene primers, actin gene primers were used as an endogenous control. The run method for qPCR included two initial hold cycles of 50° for two minutes followed by 95° for ten minutes, then 40 cycles of 95° for 15 s followed 60° for one minute.

## Figures and Tables

**Table 1:** Blast summary statistics identifying number of hits per database against the total contig count (211,808).

Database Name	Number of Hits	% of Hits	Unique Hits
Viridiplantae nr	180,762	85.34	61.699
Rice proteins	168,274	79.45	23.982
Setaria proteins	165,887	78.32	23.358
Hits to all 3 databases	157,700	74.45	N/A
Hits to at least one database	186,170	88	N/A

**Table 2:** Number of DE contigs from each method (at significance level FDR ≤ 0.05).

Comparisons	DESeq2	RSEM-EBSeq
HS vs. MS	30,159	48,920
HS vs. LS	42,325	42,550
MS vs. LS	15,867	34,556

**Table 3:** Number of differentially expressed contigs from EBSeq with multiple conditions (at FDR ≤ 0.05 or PPDE ≥ 0.95).

Comparisons		EBSeq
Pattern 1	Similar expression between HS and MS but different for LS	9122
Pattern 2	Similar expression between HS and LS but different for MS	22,601
Pattern 3	Similar expression between MS and LS but different for HS	25,576
Pattern 4	Different expression between all conditions	16,312
(Total DE contigs across all above-listed patterns)		73,611

**Table 4:** Transcript per million (TPM) expected counts per contig for each sample in triplicate for low, medium, and high samples in biological replicate.

NAME	LSBR1	LSBR2	LSBR3	MSBR1	MSBR2	MSBR3	HSBR1	HSBR2	HSBR3
tra_7509	4.97	8.57	7.7	0.93	2.33	2.63	27.83	23.96	22.89
tri_352115	194.98	213.66	264.16	55.66	253.55	275.44	0.12	0	0
tri_256589	0	0	0	2.46	4.24	2.32	0.21	0	0

**Table 5:** qPCR analysis for three primer sets derived from salt-related sequence expression data including low (LS)-, medium (MS)-, and high (HS)-salinity samples. The table details data for sample name, target name, RQ (relative quantification), RQMIN (RQ minimum), RQMAX (RQ maximum), CT (cycle threshold), CTSD (CT standard deviation), ΔCT Mean, and ΔΔCT.

Sample Name	Target Name	RQ	RQ Min	RQ Max	CT	CTSD	ΔCT Mean	ΔΔCT
LS	352115	1	0.636477	1.57115	25.34552	0.217588	−7.13879	0
MS	352115	1.816862	0.907651	3.636846	24.4925	0.53795	−8.00024	−0.86145
HS	352115	2.047153	1.063221	3.941641	23.79867	0.266774	−8.17241	−1.03362
LS	256589	1	0.345292	2.896101	35.31568	0.893261	3.630939	0
MS	256589	1.618414	0.95379	2.746165	35.42017	0.218514	2.936359	−0.69458
HS	256589	1.965519	1.051805	3.672984	34.31187	0.200397	2.656029	−0.97491
LS	7509	1	0.456056	2.192713	31.32734	0.617515	−0.35185	0
MS	7509	0.097121	0.026046	0.362148	34.99089	1.141173	3.012215	3.364066
HS	7509	0.234553	0.081096	0.678395	33.37183	0.798214	1.74016	2.092012

**Table 6:** KEGG pathway mapping.

KEGG Pathway Mapping (MS vs. LS)			
Rice KEGG-ID	Pathway	DEG_Name	Pathway Name
K12813	Spliceosome	tra_17_c0_g1_i1	Pre-mRNA-splicing factor ATP-dependent RNA helicase DHX16
K00021	Metabolic pathway/biosynthesis of secondary metabolites	tri_275973_c0_g1_i1	Hydroxymethylglutaryl-CoA reductase (NADPH)
K02703	Photosynthesis/metabolic pathways	tri_157558_c0_g1_i1	Photosystem II P680 reaction center D1 protein
K15109	Thermogenesis	tri_161409_c0_g1_i1	Solute carrier family 25 (mitochondrial
K00083	Phenylpropanoid biosynthesis	tri_182549_c0_g1_i1	cinnamyl-alcohol dehydrogenase)
K00928	Glycine, serine, and threonine metabolism/biosynthesis of amino acids	tri_24691_c0_g1_i1	Aspartate kinase
**KEGG Pathway Mapping (HS vs. LS)**			
**Rice KEGG-ID**	**Pathway**	**DEG_Name**	**Pathway Name**
K09284	AP2; AP2-like factor	tri_68476_c0_g1_i1	AP2-like factor, euAP2 lineage
K16296	Serine carboxypeptidase-like clade	tri_6216_c0_g1_i1	Serine carboxypeptidase-like clade I
K14513	MAPK signaling pathway—plant/plant hormone signal transduction	tri_244640_c0_g1_i1	Ethylene-insensitive protein 2
K15109	Thermogenesis	tri_50326_c0_g1_i1	Solute carrier family 25 (mitochondrial carnitine/acylcarnitine transporter), member 20/29
K00083	Phenylpropanoid biosynthesis	tri_234627_c0_g1_i1	Cinnamyl-alcohol dehydrogenase
**KEGG Pathway Mapping (HS vs. MS)**			
**Rice KEGG-ID**	**Pathway**	**DEG_Name**	**Pathway Name**
K00799	Glutathione metabolism	tri_200548_c0_g1_i1	Glutathione S-transferase
K00166	Valine, leucine, and isoleucine degradation/propanoate metabolism	tri_19937_c0_g1_i1	2-Oxoisovalerate dehydrogenase E1 component subunit alpha
K08913	Photosynthesis—antenna proteins/metabolic pathways	tra_19146_c0_g1_i1	Light-harvesting complex II chlorophyll a/b-binding protein 2
K04079	Protein processing in endoplasmic reticulum/PI3K-Akt signaling pathway	tra_32300_c0_g1_i1	Molecular chaperone HtpG
Kl4497	MAPK signaling pathway—plant/plant hormone signal transduction	tri_344092_c0_g1_i1	Protein phosphatase 2C

## Data Availability

The data presented in this study are available on request from the corresponding author.

## Supplementary Materials

The following supporting information can be downloaded at https://www.mdpi.com/article/10.3390/plants13142008/s1: Table S1. Realtime qPCR primers quantified in the gene expression analysis. 18S rRNA primer set obtained from Yu et al., 2019 [36]. Primers with an asterisk (*) were used in analysis with Figures and Tables published within this article. Seven additional primers designed from salt-related genes identified through the present transcriptome sequence annotation.
