# Supplementary material for: Development of a Reference Transcriptome and Identification of Differentially Expressed Genes Linked to Salt Stress in Salt Marsh Grass (Sporobolus alterniflorus) along Delaware Coastal Regions
Source: Plants (Basel). 2024 Jul 22;13(14):2008. doi: 10.3390/plants13142008 (PMC11280579; doi:10.3390/plants13142008)
Supplement: Supplementary file 1 [file plants-13-02008-s001.zip › plants-3018994-supplementary.pdf]

Table S1. Realtime qPCR primers quantified in the gene expression analysis. 18S rRNA primer set obtained from Yu et al.,2019. Primers with an asterisk (*) were used in analysis with Figures and Tables published within this article. Seven additional primers designed from salt-related genes identified through the present transcriptome sequence annotation.

**Endogenous Gene**		Amplicon size	Tm	Efficiency	Reference	
18S rRNA FWD	TGACGGAGAATTAGGGTTCGA	100bp	60	99%	Yu, Y., Zhang, G., Chen, Y. et al.	
18S rRNA REV	CCGTGTCAGGATTGGGTAATT					
**Designed Primers for qPCR**						
*tri_352115 FWD	GTACGGTTCTGCTTGCTTCT		55			
*tri_352115 REV	TTGTCTGAGTGCTCCGTTTC					
*tri_256589 FWD	CCCGCCAAATTCCACAAATC		55			
*tri_256589 REV	ACAGGAGGAGAAGGAACAGTA					
*tra_7509 FWD	GTCCCACCTCTGAACAACAA		54			
*tra_7509 REV	AACCAGCGAACGGGATTT					
LOC_Os12g39400.1 FWD	CAAGCGATGCCACTACGA		55			
LOC_Os12g39400.1 REV	ATGAGCCTGGGCTTCTTG					
LOC_Os01g50030.1 FWD	TGGAACTTGGTGCTGGAATAG		55			
LOC_Os01g50030.1 REV	CAACCTCCTCGTCTGAAAGATAC					
LOC_Os09g02214.3 FWD	GTACGGGTGGTTCTATGCTTATC		55			
LOC_Os09g02214.3 REV	GATAAATGGGATCTCGGCTAGTG					
Seita.9G518700.1 FWD	TGGTGGCGCTGTTCTATATG		55			
Seita.9G518700.1 REV	GTCACCTTCCCAACGATCAA					

Seita.3G034000.1 FWD	GCGTCCATCATTCTCCACTT		55			
Seita.3G034000.1 REV	CACCAGCAAAGAAAGCACATC					
Oropetium_06645 FWD	AGAGTGCCAACACCGTTATC		55			
Oropetium_06645 REV	AGGCAGGAATGGCTGAATAG					
Oropetium_09602 FWD	CGAGATCTACGAGTCTGTACCT		55			
Oropetium_09602 REV	CAGCTTCCTCTTTCTTCTCCTC					
